# Assessing STEM differentiation needs based on spatial ability and engagement: implications for making activities

**DOI:** 10.3389/fpsyg.2025.1545603

**Published:** 2025-08-26

**Authors:** Ellen Egeland Flø, Jørgen Hammer Smedsrud

**Affiliations:** ^1^Department of Educational Sciences, University of Oslo, Oslo, Norway; ^2^BI Norwegian Business School, Oslo, Norway; ^3^NIFU Nordic Institute for Studies of Innovation, Research and Education, Oslo, Norway

**Keywords:** spatial ability, stem, differentiation, engagement, making activities

## Abstract

**Introduction:**

Spatial ability is robustly correlated with science, technology, engineering, and mathematics (STEM) achievement, but schools have generally not differentiated with regard to this ability. Moreover, the level of involvement, focus, and motivation that students exhibit in school activities, specifically their engagement, also plays a central role in overall achievement. Therefore, this study aims to develop a STEM scale to identify differentiation needs based on spatial ability and engagement. These differentiation needs may be addressed by instructional methods such as making activities, where students design and construct physical objects. Because these activities enhance engagement, can improve spatial ability, and allow students to use their spatial skills, this study also aims to discuss the implications of using making activities for differentiation.

**Methods:**

To address these aims, 535 students from grades 5 to 10 (9–16 years) from two medium-sized suburban and semi-rural municipalities were randomly split into one exploratory and one confirmatory sample, where participants were students. An exploratory and confirmatory item response theory (IRT) approach was used for the data analysis. Correlations of the latent variable were sought for spatial ability and engagement.

**Results:**

The STEM scale demonstrated good psychometric properties, and the underlying factor of the scale correlated positively with engagement and spatial ability.

**Discussion:**

Thus, the STEM scale can be useful for educational practice by identifying students needing differentiation concerning their spatial ability level, where their level of engagement is also considered. Making activities could potentially benefit some students more than others, depending on their STEM scale scores.

## 1 Introduction

STEM education is central to preparing students to face real-world problems (Wai et al., 2009), to develop technological solutions to address such problems, and understand the technology surrounding them (Norwegian Directorate for Education and Vocational Training, 2018). Multiple countries’ policymakers have recognised this through funding of STEM-educational programmes, such as Germany, Finland, and the US (Ertl et al., 2017; Lavonen and Laaksonen, 2009; Tanenbaum, 2016) in concurrence with generally increased importance given by governments to STEM subjects (Young and Muller, 2016).

Spatial ability is a strong predictor of STEM achievement and STEM career choice (Wai et al., 2009), as well as a systematic source for individual differences in STEM learning (Bongers et al., 2020; Li and Wang, 2021). However, spatial reasoning is less emphasised in schools (Ramey et al., 2020). Most school curricula are primarily suited to students with high verbal and mathematical reasoning abilities, and a focus on spatial ability is lacking (Wai and Uttal, 2018; Wai and Worrell, 2016). In a recent study, students with high spatial ability and lower mathematical or verbal abilities had greater academic challenges and drop-out rates than students with high abilities in other domains (Lakin and Wai, 2020). This finding underlines the need for identification and differentiation based on spatial ability, because if these students were identified and differentiated for, their STEM achievement and retention rates would likely increase.

However, there is a lack of high-quality identification measures that define how to differentiate within STEM subjects for students based on their spatial ability (Kell et al., 2013). Such a measure should also include the assessment of student engagement because engagement has shown a positive relation with academic performance (Finn and Zimmer, 2012; Maxwell et al., 2017; Moubayed et al., 2020) and well-being (Gander et al., 2016), and a lack of engagement is connected to an increased risk of school drop-out (Finn and Zimmer, 2012; Piscitello et al., 2022) which is very relevant to STEM retainment. Engagement is a multifactorial term concerning students’ involvement in school, with both subject matter and social interactions (Finn and Zimmer, 2012; Groccia, 2018). The most common factors are behavioural engagement (e.g., participation, interaction, collaboration, completion of learning activities), cognitive engagement (e.g., motivation and effort to learn, reflection), and affective engagement (e.g., attitudes and feelings directed at teachers, peers, and content) (Fredricks et al., 2016; Salas-Pilco et al., 2022).

Both engagement and spatial ability are essential for STEM achievement and for how advanced material students are willing and able to work with. So, when identifying student differentiation needs in STEM, basing the assessment on engagement and spatial ability is pertinent. Furthermore, because proper differentiation and teaching methodology can be beneficial for student learning and motivation (Amerstorfer and Münster-Kistner, 2021; Schmidt et al., 2016; Kong, 2021; Smedsrud et al., 2024; Zeng et al., 2016), the identification of differentiation needs is important for improving future STEM education and outcomes, in particular for underserved student groups (e.g., with higher spatial ability than verbal/mathematical abilities).

“Basic science indicates that students throughout the ability range could profit from spatial ability assessments and the provision of educational opportunities aimed at developing spatial ability” (Wai et al., 2009, p. 818)

Furthermore, low spatial ability can form a barrier between novice and expert STEM achievement (Dawson, 2019), because spatial ability is central for STEM learning for novices. After more subject-specific expertise develops, drawing on specific knowledge tends to replace the need for general spatial reasoning, thus reducing the reliance on spatial abilities (Dawson, 2019). Accordingly, low spatial ability can act as a barrier due to loss of learning and motivation for a STEM novice with lower spatial ability, which never reaches the expertice level where spatial ability no longer matters as much. Furthermore, females generally score somewhat lower than males on spatial ability tests (Levine et al., 2016), turning this into a potential gender barrier as women are underrepresented in STEM careers (González-Pérez et al., 2020).

Fortunately, spatial ability is malleable (Uttal et al., 2013), and numerous interventions have been implemented for improving it. These interventions have traditionally focused on developing specific spatial abilities, often through video or digital gaming environments (Uttal et al., 2013). More recently, the focus has shifted towards interventions that may develop spatial abilities concurrently with STEM subject learning (Munoz-Rubke et al., 2021; Plummer et al., 2022) to avoid loss of valuable school lesson time (Stieff et al., 2018). One of the most promising avenues for such interventions is making activities where students design and make STEM-relevant physical artefacts. Several studies on such interventions have found that students use and improve their spatial abilities/skills (Bhaduri et al., 2019; Falloon et al., 2020; Guo et al., 2022; Mercan and Kandır, 2022; Munoz-Rubke et al., 2021; Ramey et al., 2020; Simpson and Kastberg, 2022; Smith, 2018; Trumble and Dailey, 2019), in addition to increasing their engagement and enjoyment (Konstantinou et al., 2021).

Spatial ability is central to STEM learning, and spatial ability-based differentiation may facilitate increased STEM learning and engagement. Thus, the current study aims to develop a scale to identify differentiation needs based on individual differences in spatial ability and student engagement. To address the differentiation needs identified by this scale, we discuss the potential for making activities, as these activities are STEM-relevant, increase student engagement, and show promise regarding spatial ability improvement.

### 1.1 Spatial ability

Spatial ability can be defined in several ways, such as a “skill in representing, transforming, generating, and recalling symbolic, non-linguistic information” (Linn and Petersen, 1985, p. 1482) or “the ability to generate, retain, retrieve, and transform well-structured visual images” (Lohman, 2013, p. 98).

Spatial ability originated from psychometric testing as a spatial factor separate from general intelligence (Mohler, 2008; Malanchini et al., 2020). This spatial factor was divided into several key aspects such as spatial visualisation, mental rotation, perspective taking, and embedding/field independence (Ramful et al., 2017; Hawes et al., 2015). Internal imaging/spatial visualisation is tied to visualising different objects and how they are connected, which is also closely tied to doing the corresponding practical task – such as paper folding (Castro-Alonso and Atit, 2019). Mental rotation in 2D or 3D deals with rotating shapes in the mind to see how they look from other angles (Castro-Alonso and Atit, 2019), while perspective taking is connected to visualising situations from other points of view and putting oneself in another position (rotating or moving the visualised self) (Kozhevnikov and Hegarty, 2001). Lastly, embedding/field independence is related to identifying the relevant visual information and is tied to the coherence principle – that higher spatial ability makes it easier to overlook redundant information (Castro-Alonso, 2019).

There have been varied approaches to understanding spatial abilities, each providing a unique contribution (psychometric, developmental, differential, and information processing), making it clear that this is a multifaceted set of complex cognitive abilities (Mohler, 2008). Accordingly, there has been a development towards multidimensional models for spatial ability, with a set of separate but correlated skills. The factors of spatial visualisation, mental rotation, perspective taking, and embedding/field independence arose from the bottom-up factorial analysis of cognitive tests; however, a more top-down theoretical approach has been sought to increase understanding of the cognitive processes involved (Buckley et al., 2018). From a cognitive neuroscience point of view, a theoretical quadrant model proposed by Uttal and colleagues (Uttal et al., 2013; Newcombe and Shipley, 2015) argues that the construct can be divided along two orthogonal axes, namely extrinsic/intrinsic and static/dynamic. The intrinsic/extrinsic axis denotes whether the spatial information refers to an individual object or relations between several objects or frames of reference, and the static/dynamic axis denotes whether there is motion involved (Uttal et al., 2013).

The most common spatial ability tests mainly assess the intrinsic-dynamic quadrant, which does not represent what is needed in real-world problem-solving contexts (Ramey et al., 2020). More recent developments argue for including all quadrants in assessment (Ramey et al., 2020), which the developed scale seeks to do. Furthermore, the different spatial ability factors (spatial visualisation, mental rotation, perspective taking, and embedding/field independence) have demonstrated varying significance for different STEM areas (Buckley et al., 2022). To address these results in our scale, we intend it to be one-dimensional, capture the commonality between the four factors, and be broad enough to be useful for different STEM areas.

#### 1.1.1 Malleability and making

Increasing spatial ability through interventions is possible, with Uttal et al. (2013) review identifying an overall effect size of 0.47. Their meta-analysis investigated interventions that aimed at increasing spatial ability through video games (12%), courses (21%), and spatial task training (67%). Moreover, transfer effects of these interventions were present, as training in spatial ability within one quadrant had positive effects on spatial abilities within another quadrant (Uttal et al., 2013). Furthermore, a recent study found that a curriculum designed to improve spatial ability also improved verbal reasoning and that spatial cognition gains predicted and mediated verbal reasoning improvement (Cortes et al., 2022). These findings were further substantiated by fMRI-observed neural changes that predicted and mediated learning transfer. The results also support the in-school development of spatial abilities (Cortes et al., 2022), which are currently lacking (National Research Council and Geographical Sciences Committee, 2005; Newcombe, 2017; Wai and Uttal, 2018) and that such improvements in spatial ability may also be transferable to verbal reasoning.

Recent research on spatial ability has investigated whether classroom-based interventions can increase students’ spatial ability with a more naturalistic rather than laboratory approach. Several of these studies used makerspace or making/engineering activities in a classroom setting, where students design and make STEM-relevant artefacts, to investigate the presence/need for spatial ability and its malleability (Bhaduri et al., 2019; Falloon et al., 2020; Guo et al., 2022; Mercan and Kandır, 2022; Munoz-Rubke et al., 2021; Ramey et al., 2020; Simpson and Kastberg, 2022; Smith, 2018; Trumble and Dailey, 2019).

Observational and correlational studies have been conducted to identify a connection between making activities and spatial ability. Recent studies identified spatial reasoning in making activities in non-formal and formal educational settings, respectively (Simpson and Kastberg, 2022; Falloon et al., 2020). Falloon et al. (2020) found several instances of young students (5–8 years) needing/using mathematical knowledge in a makerspace program. These instances were connected to a 3D app to improve several skills, e.g., spatial awareness. In making activities, 3-D design and printing are prominent, and several studies have investigated the relation between such activities and spatial skills. One study’s findings highlight that students negotiate visualisation skills as they move between digital and non-digital technologies by manipulation through digital 3-D modelling, mental rotation, and mental representation (Smith, 2018). Whereas another study investigating a 3D modelling/printing summer school found that when curricular coherence is emphasised as a design goal and providing students with multiple avenues for engaging in 3D modelling, it can help to provide youth with opportunities to develop their spatial thinking skills (Bhaduri et al., 2019).

Furthermore, Ramey et al. (2020) found that students participating in school-based making activities engaged in frequent and diverse spatial reasoning. The students’ spatial reasoning developed over time and led to learning. The most frequent quadrant (according to the quadrant model) was extrinsic-static skills (57%), followed by intrinsic-static (24%), extrinsic-dynamic (11%), and intrinsic-dynamic skills (8%) (Ramey et al., 2020). It is important to consider that most spatial ability tests primarily measure intrinsic-dynamic skills, such as 2D/3D rotation and paper-cutting tests. However, it is necessary to have a more comprehensive method of identifying students’ spatial abilities that includes other relevant aspects in real-world problem-solving situations. Therefore, the current study aims to create a scale that includes items from all four spatial ability quadrants.

Moreover, several recent studies have investigated whether making activities can improve spatial ability, with promising results. Trumble and Dailey (2019) found that students participating in a STEM camp driven by maker pedagogy improved their spatial abilities significantly. Later studies included control groups in their design and found that 3D design can improve students’ spatial ability and that there was no significant difference in how considerable the improvement was for high/low initial spatial ability, i.e., both groups improved about the same (Guo et al., 2022). Other controlled studies identified improvement in visual–spatial reasoning skills in children (5–8 y) after a makerspace education program (Mercan and Kandır, 2022) and that making activities involving mechanical problem-solving improved students’ spatial ability by directly engaging with STEM content (Munoz-Rubke et al., 2021).

Because schools underemphasise spatial ability (Ramey et al., 2020), spatial ability is not stimulated to be developed as mathematical or verbal abilities are stimulated through specific mathematics and language subject teaching. This lack of focus may be one of the reasons why spatial ability is found to be so malleable through specific interventions, because spatial ability is not developed in schools. So that when comparing spatial ability gains between students in an intervention to students only participating in ordinary schooling, the difference between the two groups is large. There is a need to address how to stimulate the development of spatial abilities through school-based interventions, due to this untapped potential. Such an intervention could be making activities that demonstrate the potential for increasing spatial ability in addition to engagement (Konstantinou et al., 2021) and learning (Flø, n.d., In review), as will be discussed in section 5.2.2.

### 1.2 Spatial ability connected to STEM

“Visual–spatial ability is a multifaceted component of intelligence that has predictive validity for future achievement in STEM occupations” (Andersen, 2014, p.114). In a recent study, the combination of mental folding (intrinsic-dynamic) and spatial scaling (extrinsic-static) accounted for 8% of the variance in science scores for children aged 7–11 years (Hodgkiss et al., 2018). This indicates the relationship between spatial abilities and STEM holds for young pupils using the quadrant model, namely the division of spatial ability into two axes, i.e., extrinsic/intrinsic and static/dynamic (Hodgkiss et al., 2018).

This connection is further substantiated by analysing decades of longitudinal research, showing that spatial ability has a robust influence on STEM domains (Wai et al., 2009). Spatial abilities add incremental validity beyond SAT-Mathematical and SAT-Verbal scores in predicting selected STEM criteria such as level of STEM education and STEM career choice (Wai et al., 2009), thus implying that students with different ability profiles, but relatively higher spatial abilities, could benefit from identification and intervention based on spatial ability criteria. The size of the association between different STEM fields and spatial ability is generally not apparent. However, a moderate association between spatial and mathematical skills independent of grade level and gender (r = 0.36) has been identified (Atit et al., 2021). Additionally, fluid reasoning and verbal skills mediated the relationship, but a unique relation between the spatial and mathematical skills remained. However, because this study was correlational, no conclusions on causality could be made. One study investigating the causality between improving spatial ability and STEM improvement (i.e., transfer from spatial ability training to STEM achievement) found that college students who received a spatial skills training course improved their STEM outcomes as well as showed increased female retention in engineering compared to students who did not receive the course (Sorby et al., 2018).

A recent review had mixed findings regarding the impact of spatial ability on science achievement (Chen et al., 2020). They argue that there seems to be an expert/novice effect where the experts no longer require spatial skills to solve specific tasks but instead use domain-specific knowledge. In contrast, the novices have yet to learn domain-specific knowledge and are thus more dependent on the general spatial skills needed to reason their way to a solution. It is further argued that certain STEM areas are only connected to a subset of spatial abilities (Chen et al., 2020), as also suggested by a cognitive-neuroscience-informed study where behavioural measures of spatial abilities demonstrated different relations to different STEM areas (Li and Wang, 2021). Because the scale we wish to develop is meant to be used as broadly as possible for non-expert students to aid teachers with identifying differentiation needs, a domain-general spatial ability approach is sought when designing the scale items.

### 1.3 Differentiation and engagement

A common understanding that cognitive ability and capacity positively correlate with engagement and academic achievement exists (Lakin and Wai, 2020). However, some recent studies challenge this assumption. A study by Smedsrud (2018) suggests that the opposite can be true for the higher end of the cognitive spectrum. Thus, ability level and cognitive capacity do not necessarily correlate with engagement and academic achievement (Lakin and Wai, 2020). Some children may need to receive early academic challenges and opportunities for more advanced levels of understanding, to experience engagement and to reveal their potential to the environment, and not to face an increased risk of underachieving compared to their potential (Smedsrud et al., 2024; Steenbergen-Hu et al., 2020). Studies also suggest that teachers find it difficult to differentiate their instructions for high-achieving students compared with the general student group (Brevik et al., 2018; Rotigel and Fello, 2004), and thus, these students do not always receive proper cognitive challenges in the regular classroom (Diezmann, 2005), and they report less support by their teachers than lower ability students (Smedsrud, 2018).

Early identification of students’ optimal differentiation levels would enable teachers to adapt their instruction to such students. In this way, fewer students will potentially underachieve, thus increasing the number of students who fulfil their potential. Additionally, as a student’s engagement is often linked to feelings of mastery, which emerge when given suitably challenging tasks, we argue that early identification could lead to greater STEM recruitment if students receive properly differentiated education in STEM subjects.

Such engagement outcomes have been the focus of much makerspace research (Mersand, 2021), and findings indicate that making and makerspace activities generally enhance engagement (Konstantinou et al., 2021). Moreover, there is an interaction between spatial skills and engagement concerning achievement, as a recent study found that students’ spatial skills significantly interact with motivation (an aspect of engagement) to predict mathematics performance for middle school students (Atit et al., 2020). Their findings underline that achievement is not only due to ability, and engagement is also central.

Thus, students with higher levels of engagement can be more motivated to work with more advanced STEM material, and vice versa, than their spatial ability level would suggest. We wish to incorporate this aspect into the scale and hypothesise that it will correlate with engagement as measured by the positive school engagement behaviour scale described in section 3.

There is a solid link between spatial ability and STEM achievement and career choice, but there is also a need for more spatial ability learning opportunities in school. Because makerspace activities include spatial thinking and skills and increased spatial ability, in addition to increasing student engagement, such activities may be beneficial for STEM achievement and recruitment purposes. As proper student differentiation is linked to increased motivation and engagement, the current study aims to develop a STEM scale to identify individual differentiation needs based on students’ spatial ability and engagement levels in combination with their explicit STEM knowledge level. This is done to improve STEM learning, recruitment, and retention and to inform the instructional practice of making activities in STEM lessons in school. In pursuit of this aim, we seek to answer the following research questions.

*RQ1:* Does the proposed self-report STEM scale have acceptable psychometric properties in terms of (a) internal structure, (b) difficulty, (c) measurement precision, and (d) measurement invariance with respect to gender?

*RQ2:* How does the underlying general factor of the STEM scale relate to spatial ability (intrinsic-dynamic) and self-reported school engagement behaviour, and what are the implications for the educational practice of making activities?

## 2 Materials and methods

### 2.1 STEM scale design

We started by generating 22 qualitative items describing specific STEM skills based on the classical sub-factors of spatial ability, namely spatial visualisation, mental rotation, perspective taking, and embedding/field independence (Ramful et al., 2017; Hawes et al., 2015). These 22 items can be found in Appendix A and consist of STEM skills regarding:

- Spatial visualisation: Connecting graphs to functions and fractions’ relation to the number line.
- Mental rotation: Checking whether two molecules are mirror images or not, also tied to constructing objects and putting things together (design-based STEM).
- Perspective taking: Reading maps, following directions, or understanding movement in mechanical systems and anatomy.
- Embedding/field independence: Finding relevant information in graphs and tables or multimodal representations.

Two independent experts were recruited based on their extended experience within spatial ability and STEM education and research to evaluate the items for theoretical suitability. All 22 items were approved by the experts. However, the exploratory multidimensional IRT calculations assessing dimensionality of the scale demonstrated that there was no single factor for the 22 items. To obtain the aim of constructing a one-dimensional scale tapping into the general spatial ability construct, the experts suggested a re-evaluation of the items with regard to theory. The authors evaluated all items theoretically, this time concerning a more explicit connection between spatial ability and STEM skills, which reduced the items to 10. This reduction in item number was then approved by the experts.

We also considered a study that combined astronomy and spatial ability, in which an intervention focusing on lunar phases and the seasons improved students’ perspective-taking and spatial thinking within astronomy (Plummer et al., 2022) when constructing items. Due to this connection, we included seasons and lunar astronomy items in the preliminary scale. However, exploratory psychometric calculations excluded the seasonal item as it was too similar to the lunar item. Such redundant items should be eliminated in the development process so that the final scale contains sufficiently distinct items and does not artificially inflate reliability levels (Carpenter, 2018). Since we wished to develop a one-dimensional scale to cover the broader construct of spatial ability translated into STEM skills, the items needed to have about the same amount of similarities/differences to each other, i.e., all items needed to share a similar amount of variance with the remaining other items. The resulting scale thus consisted of 9 items.

These 9 items can be placed within the quadrant model framework (Uttal et al., 2013; Newcombe and Shipley, 2015); see Table 1. We found that all four quadrants were represented by these 9 items, as opposed to the classical pencil and paper tests, which mainly focus on the intrinsic-dynamic quadrant, which is not representative of the diversity of spatial skills needed in real-world settings (Ramey et al., 2020). This process of operationalising spatial ability into qualitative statements connected to STEM achievement and further refining the selection of items based on exploratory calculations and theoretical refinement caused the content to move into a broader construct in line with real-world classroom applications. This is in line with our aim of creating a unidimensional scale for STEM differentiation needs based on a broad spatial ability construct, rather than a spatial ability test to directly assess students’ skill level for each quadrant.

**Table 1:** An overview of the items in the STEM scale and which of the quadrants they belong to.

Items	Quadrant
1. I am good at understanding coordinate systems.	Intrinsic-static
2. I am good at finding what is important in tables or graphs.	Intrinsic-static
3. It is easy for me to find what is important in a text.	Intrinsic-static
4. I understand what different molecules look like.	Intrinsic-dynamic
5. I can easily understand the connection between a fraction and the number line.	Extrinsic-static
6. I am good at understanding the connection between a graph and its corresponding formula.	Extrinsic-static
7. I am good at making the right connections in electrical circuits.	Extrinsic-dynamic
8. I understand why the backside of the moon is always facing away from the Earth.	Extrinsic-dynamic
9. I am good at understanding what we are supposed to do in science experiments.	All categories, but dependent on the specific experiment

The participants answered the items on a five-point Likert scale, with the categories “Very poor fit,” “Somewhat poor fit,” “Neutral,” “Somewhat good fit,” and “Very good fit.” These categories were initially coded as 1, 2, 3, 4, and 5, respectively.

### 2.2 Other measures

The spatial reasoning instrument (SRI) (Ramful et al., 2017) was designed to assess spatial reasoning abilities in children. The SRI consists of 30 multiple-choice tasks aimed at measuring different aspects of spatial ability. These aspects involve mental rotation, spatial visualisation, spatial orientation, and spatial relations (Ramful et al., 2017). The latter is a description of the relations between the three former. The scale was constructed to assess middle school students’ spatial ability and shows robust psychometric properties in terms of validity and reliability (Ramful et al., 2017).

The Positive School Engagement Behaviour (PSEB) subscale aims to identify students’ positive engagement behaviour in school (Skinner et al., 2009). It has been validated for use in a Swedish school context (Ritoša et al., 2020), which is quite similar to the Norwegian context and language in the current study context. The PSEB subscale consists of five items, which are scored on a four-point Likert scale.

### 2.3 Participants

The first sample consisted of 293 students in grades 5 to 10 (9–16 years) from a medium-sized semi-rural municipality with one small city in the southwest of Norway, with a population of approximately 20,000, see Table 2. The second sample consisted of 242 students in the same age group from a medium-sized suburban municipality in the southeast of Norway with a population of approximately 40,000, see Table 3. The municipalities were recruited based on including rural, urban, and suburban areas that most closely represented the corresponding distribution for the whole country. Students were recruited through their respective municipalities, and whole randomised classes participated. This was done to ensure the participation of students of all ability levels, rather than a non-random selection of students. The two samples were similar in size and age/class distribution, but any differences were removed by pooling both samples and then randomly splitting them into two groups to perform an exploratory and confirmatory model estimation.

**Table 2:** The first student sample by self-reported gender and grade.

Number of participants	Male	Female	Unidentified	Grade 5	Grade 6	Grade 7	Grade 8	Grade 9	Grade 10
293	145	131	17	63	55	26	71	47	31

**Table 3:** The second student sample by self-reported gender and grade.

Number of participants	Male	Female	Unidentified	Grade 5	Grade 6	Grade 7	Grade 8	Grade 9	Grade 10
242	113	113	16	22	20	19	70	109	2

We chose to include an age range which covers a period of much cognitive development. However, this may not be the case with regard to spatial ability because of the lack of spatial ability teaching in schools (Wai and Uttal, 2018; Wai and Worrell, 2016), which may hinder its further development during the adolescent years. This assumption will be checked through a comparison of the descriptive statistics for the SRI for this study’s sample with student groups encountering a ceiling effect (Harris et al., 2021) and those who do not (Lowrie et al., 2017). Moreover, the reading level changes during this period and is important to incorporate in scale development (Gorsuch and Venable, 1983), which was done through keeping items short and fit for the youngest participants while still being relevant for the older participants.

### 2.4 Data collection

Data was collected through an electronic form of the STEM scale, and all items were mandatory so that there were no missing data in our dataset.

### 2.5 Data analysis

We used R version 4.3.1 (R Core Team, 2023) with R studio version 2023.12.1 for the statistical analysis and estimated the IRT models with the R package mirt (Chalmers, 2012).

#### 2.5.1 Descriptive statistics

We used item-to-total score Pearson correlations and item mean scores as an initial screening of item quality. Items with an item-to-total score Pearson correlation lower than 0.3 were selected for further review. We furthermore estimated Cronbach’s α to obtain a lower-bound estimate of the reliability of the sum scores.

#### 2.5.2 Dimensionality assessment

We evaluated dimensionality by randomly splitting the entire data set into two equal-sized parts: one part for an exploratory analysis of dimensionality and one part for a confirmatory analysis based on the exploratory results. The data splitting resulted in sample A with 268 respondents and sample B with 267 respondents. Initial analysis revealed that the lowest categories were rarely endorsed, and we thus combined the two lowest categories into one. With the recoded data, we performed a full-information exploratory item response theory analysis with sample A by fitting unrestricted item response theory models with one and two dimensions and selecting the model with the lowest Bayesian Information Criteria (BIC) (Cho et al., 2016; Kim et al., 2019).

#### 2.5.3 Item response theory estimation and evaluation

Similar to confirmatory factor analysis models, IRT views the construct of interest (i.e., STEM differentiation level) as a latent variable that cannot be directly observed. IRT models assume that the probability of a participant endorsing an item is a function of two sets of parameters. The first is their position on the STEM differentiation level continuum (person parameter), and the second is each item’s properties (item parameters).

Based on the exploratory IRT analysis results, we fitted a confirmatory IRT model with sample B and evaluated the model and item fit. Model fit was assessed with the M2 hypothesis test (Liu et al., 2016), the root mean square error of approximation (RMSEA), and the standardised root mean squared residual (SRMSR) fit statistics. We used a significance level of 0.05 for the M2 hypothesis test of absolute fit and the cut-off values RMSEA < 0.06 and SRMSR < 0.08 for good approximate fit (Cai and Monroe, 2014; Maydeu-Olivares and Joe, 2006). Item fit evaluation was done with Sχ^2^ hypothesis tests (Kang and Chen, 2008) with a Bonferroni-adjusted overall significance level of 0.05 and by graphical evaluation of model-based and empirical item category response functions.

#### 2.5.4 Psychometric properties of the STEM scale

We evaluated the measurement properties of the scale using the estimated item response theory model. We estimated the reliability of the sum scores and IRT ability scores with model-based reliability coefficients (Cheng et al., 2012). The scale difficulty was evaluated with the expected test score function, which indicates the average sum scores for given values of the latent variable. The measurement precision of the scale was inferred from the test information function.

#### 2.5.5 Measurement invariance

We investigated measurement invariance for gender by first fitting a configural model without any restrictions across groups and evaluating the model fit. We then fitted a semi-constrained model with all item intercepts set equal between the groups. This model was then compared against the configural model with a likelihood ratio test and a significance level of 0.05. We then fitted a fully constrained model with all item slopes and intercepts set equal between the groups. This model was then compared against the semi-constrained model with a likelihood ratio test and a significance level of 0.05.

#### 2.5.6 Correlations with external variables

To obtain a clear interpretation of the STEM scale scores, we estimated Pearson correlations between the STEM scale latent ability scores, the spatial reasoning instrument (SRI) latent ability scores, and the positive school engagement behaviour (PSEB) sum scores. We adjusted for unreliability with respect to the PSEB sum scores by utilising the sum score reliability coefficient, Cronbach’s alpha.

## 3 Results

None of the nine items of the STEM scale demonstrated a point biserial item-total correlation below 0.3. Thus, no items were removed from further analysis. We employed an exploratory dimensionality analysis where a fully unrestricted graded response unidimensional IRT model was estimated. To check for unidimensionality, a graded response model with two dimensions was also estimated and compared to the unidimensional model. According to the BIC, the best model was the unidimensional model (BIC = 5,715) compared to the two-dimensional model (BIC = 5,966).

We further inspected visual item fit by trace plots to evaluate if categories should be collapsed to better fit the data. The plots demonstrated substantial overlaps between the first two categories. Therefore, it was decided to collapse the first two categories into only one, as there was no theoretical reason to keep all five categories. The two combined categories were named “Very poor fit” and “Somewhat poor fit” and were coded as 1. The other categories were named “Neutral,” “Somewhat good fit,” and “Very good fit,” and were coded as 2, 3, and 4, respectively.

The confirmatory model evaluation also collapsed the first two categories into one before the computation of the item mean, *M* = 21.4, standard deviation, SD = 6.46, and Cronbach’s *α* = 0.88. Item mean and item-total score correlations are given in Table 4.

**Table 4:** The item means, item-total score correlation (pBIS), and internal consistency (*α*) if the item is deleted.

Item	Item mean	pBIS	α if deleted
1	2.48	0.64	0.86
2	2.59	0.61	0.87
3	2.14	0.68	0.86
4	2.15	0.65	0.86
5	2.12	0.53	0.87
6	2.43	0.53	0.87
7	2.33	0.70	0.86
8	2.63	0.66	0.86
9	2.51	0.61	0.87

Furthermore, we performed a model evaluation based on the model from the first sample with a confirmatory analysis. The unidimensional model from the exploratory analysis was applied to the second dataset from the combined sample. The assessed model-data fit by the M2-statistic, provided the values RMSEA = 0.031 [90% CI = (0, 0.079)], SRMR = 0.052, TLI = 0.98 and CFI = 0.99, demonstrating good fit (Maydeu-Olivares, 2013). The equality of model versus data sum score distribution was estimated, resulting in a value of 0.88, which demonstrates an acceptable fit (Maydeu-Olivares, 2013). The estimated reliability of the EAP (expected a posteriori) ability estimates were also calculated (Bock and Mislevy, 1982), resulting in an acceptable value of 0.89 (Maydeu-Olivares, 2013).

The S-*χ*^2^ test of item fit was performed to evaluate the fit of the individual items, where the Bonferroni corrected value of the probability was *p* = 0.0056 (for a confidence level of 5%). This value is very conservative, so we considered any items with *p* > 0.007 to demonstrate adequate fit. As shown in Table 5, all items demonstrated adequate fit.

**Table 5:** The results of the S-χ^2^ test of item fit.

Item	$S-\chi^{2}$	$S-\chi^{2}$ probability
1	39.37	0.281
2	42.38	0.127
3	37.91	0.218
4	27.25	0.660
5	40.59	0.171
6	52.74	0.147
7	33.49	0.218
8	23.66	0.699
9	38.31	0.322

**Figure 1:**
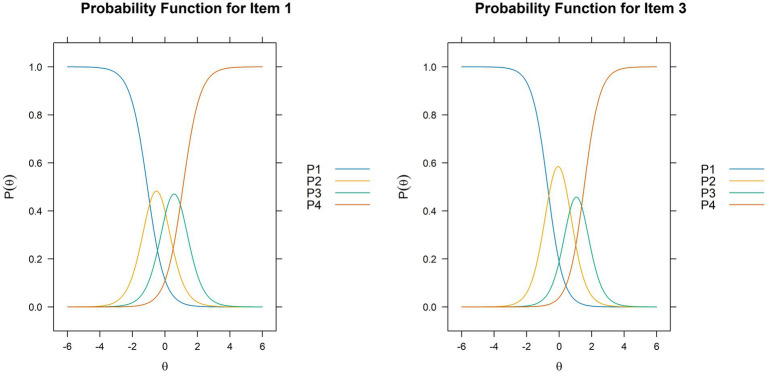
Examples of item trace curves are shown for items 1 and 3. The curves show the probability for endorsing one category for each item based on the value of the underlying trait, θ, assessed by the STEM scale.

We then visually evaluated item fit by inspecting item trace plots to check for non-overlap in categories, and no overlaps were identified. Examples of item trace curves are shown in Figure 1.

**Figure 2:**
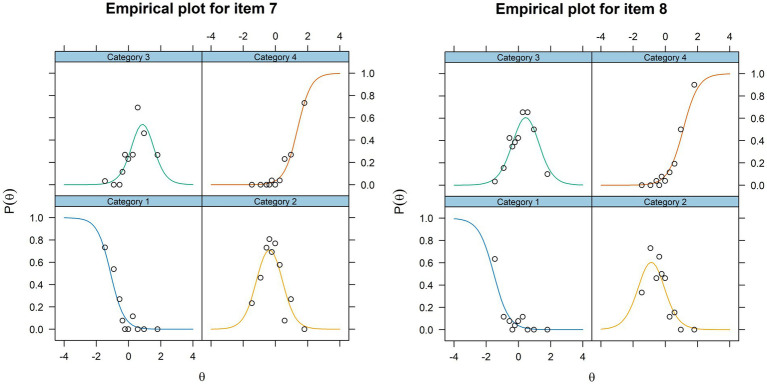
Examples of empirical plots are shown for items 7 and 8. The plots are visual impressions of how close the empirical data points are to the theoretically estimated model for each answer category for each item based on the value of the underlying trait, θ, assessed by the STEM scale.

Further, item fit was inspected visually by making empirical plots for each item, as shown in Figure 2. No items demonstrated poor fit due to large differences between the model estimated probability curves and the empirical data.

To evaluate differential item functioning and check for invariance with regard to gender, we used the Lavaan package version 0.6.16 because the IRT analysis with two groups did not converge for the fully unrestricted model. First, we estimated a configural model (i.e., all parameters estimated freely in both gender groups), model 1. This was compared to model 2, which was estimated with restricted intercepts between the two groups by a likelihood-ratio test. The test resulted in a p-value = 0.95, demonstrating metric invariance for gender. At last, model 2 was compared to model 3 with a likelihood-ratio test, estimated with restricted intercepts and slopes. This test resulted in a p-value = 0.51, also demonstrating scalar invariance for gender.

**Figure 3:**
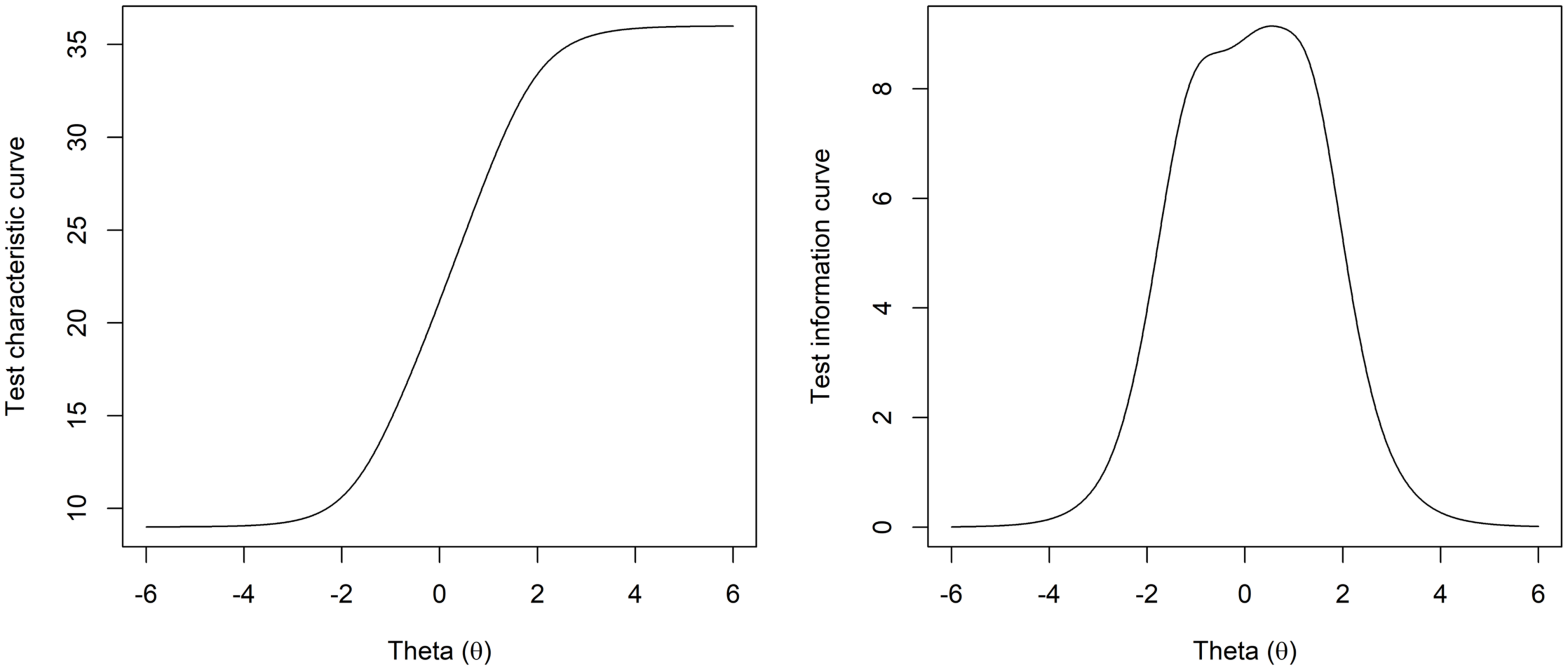
The test characteristic curve (i.e., the expected test score function) and the test information curve for the STEM scale, where Theta (*θ*) is the normalised latent variable for the ability level assessed by the STEM scale.

The test characteristic curve and the test information curve were computed and are shown in Figure 3.

The SRI-scale (Ramful et al., 2017) for spatial ability estimation produced a mean value of *M* = 15.35 out of 30 possible points, standard deviation of SD = 5.72, and internal consistency of α = 0.83. These mean and SD values were lower than for a group of Australian 11-year-olds (Lowrie et al., 2017), which indicate that ceiling effects were not an issue even though the age range in the current study was quite large. A unidimensional 2PL model fit the data best based on likelihood ratio tests (based on lowest BIC indices) of 2PL models with one and two dimensions, as well as 3PL models with one and two dimensions, which did not converge. The assessed model-data fit by the M2-statistic, provided the values RMSEA = 0.022 [CI = (0.008, 0.031)], SRMSR = 0.053, TLI = 0.98 and CFI = 0.98, demonstrating a good fit (Maydeu-Olivares, 2013).

We found the Pearson correlation of the latent variables (EAP) estimated from the best-fitting models for both the STEM scale and the SRI. This resulted in a correlation of 0.28 (*p* = 1.4e-06).

The positive school behaviour engagement scale with a 4-point Likert-type scale ranging from 1 (not at all true) to 4 (very true) (Skinner et al., 2009) produced a mean value of *M* = 13.62 out of 20 possible points, SD = 3.69, and Cronbach’s α = 0.83. No suitable measurement models were identified by either IRT or factor analysis, causing us to use latent scores (EAP estimated) for the STEM scale and sum scores for the engagement scale for correlation check, correcting for attenuation only for the engagement scale. This resulted in a correlation of 0.51 when corrected for attenuation and 0.46 when not corrected for attenuation (*p* < 2.2e-16).

## 4 Discussion

We address the psychometric properties of the STEM scale first (RQ1) before we go on to issues relating to construct validity and implications for educational practice (RQ2).

### 4.1 Research question 1

Does the proposed self-report STEM scale have acceptable psychometric properties in terms of (a) internal structure, (b) difficulty, (c) measurement precision, and (d) measurement invariance with respect to gender?

The internal structure measures how well the individual items and their relationships adhere to the description of the underlying construct being measured (Rios and Wells, 2014). We assessed this in several ways, beginning with Cronbach’s a, which is a measure of the proportion of the latent variable variance to total score variance (Crocker and Algina, 1986) and the IRT equivalent measure of a model-based reliability coefficient, namely the expected a-posteriori reliability (Brown, 2018). The reliability coefficients were 0.88 and 0.89, considered acceptable, although over 0.90 would be preferable (Boateng et al., 2018). However, according to Carpenter (2018), very high reliability coefficients may indicate that individual items in the scale are too similar so that the items do not tap into distinct aspects of the underlying construct, and the resulting scale describes a very narrow interpretation of the construct. Because we wanted to avoid a very narrow interpretation of spatial ability, to include items from all four quadrants of the quadrant model (Uttal et al., 2013; Newcombe and Shipley, 2015), these reliability coefficient values indicate a sufficiently broad construct being assessed by the STEM scale.

Another important aspect of internal structure assessment is how well the individual items fit with the other items and the overall measurement model. To assess whether each item fits in with the other items in the scale, we inspected their classical item-total correlations, which examine to what extent the items tap into the same construct (Boateng et al., 2018). These values should be at least 0.30 (Boateng et al., 2018). The STEM scale’s item-total correlations were all in the range of 0.53–0.70. Having very similar item-total correlations across all items can ensure that the underlying latent construct is one-dimensional in an IRT analysis and that all items have similar relevance for the construct. However, if these values are all similar but very high, there is a risk of inflating the reliability coefficients by measuring a construct that is too narrow. From the item-total correlation values in our analysis, it seems likely that the need for balancing these correlations being similar, yet not too high, was fulfilled, as the reliability coefficients indicated a broader one-dimensional underlying construct. We identified the STEM scale as one-dimensional, implying that all nine items tap into one broad underlying construct.

Lastly, to assess how well the individual items fit with the estimated IRT model, we did a visual inspection of the item trace curves, which show the logistically modelled relationship between the level of the latent construct of the scale and the probability of endorsing each of the alternative answers (Edelen and Reeve, 2007), and the empirical plots which are a logistic parallel to scatter plots for linear regression (Kalinowski, 2019). In addition, we performed an S-*χ*^2^ test in which each item was checked for functioning consistent with the pattern predicted by the estimated IRT model (Kang and Chen, 2011). Because all these assessments yielded acceptable results, the internal structure of the STEM scale was deemed satisfactory.

The difficulty of the STEM scale was assessed through the expected test score function in Figure 3, which indicates the average test sum scores for different levels of the latent variable, i.e., how high the ability level needs to be to achieve a specific score (Edelen and Reeve, 2007). Measurement precision was assessed through the test information curve, which is a plot of how informative the test is based on the ability level of those tested, and this is inversely related to the standard error in such a way that estimated scores are most reliable when test information is high (Hambleton and Cook, 1977). Both plots in Figure 3 indicate that the STEM scale is most useful to identify students who are not very far below or above the mean, i.e., ±2SD (which equals 95% of all students). Both curves are, however, somewhat shifted to the right of the mean so that the scale provides a more precise score estimation of above-average than below-average scores. Nonetheless, this effect is minimal, and we argue that it does not detract from the scale’s usefulness in identifying students with below mean scores.

In invariance testing, the goal is to compare whether a scale or items in the scale are understood or endorsed differently across different groups (Boateng et al., 2018). If there is both metric and scalar measurement invariance, the groups can be compared with regard to the latent variable using the scale in question (Rios and Wells, 2014). Because we identified both metric and scalar measurement invariance with respect to gender, the STEM scale can be used to compare male and female scores regarding their need for differentiation level in STEM teaching.

### 4.2 Research question 2

How does the underlying general factor of the STEM scale relate to spatial ability (intrinsic-dynamic) and self-reported school engagement behaviour, and what are the implications for the educational practice of making activities?

#### 4.2.1 Spatial ability, engagement, and the STEM scale

We aimed to develop a one-dimensional scale based on a broad understanding of spatial ability where all four quadrants of the quadrant model were represented (Uttal et al., 2013; Newcombe and Shipley, 2015). The results show that the nine STEM scale items cover all four quadrants (as reported in Table 1) and are best represented by only one dimension. Moreover, different spatial ability instruments within the same quadrant (but not measuring the same aspect of spatial ability) typically correlate between 0.22 and 0.45 and upwards based on seven studies with multiple correlations reported by Castro-Alonso and Atit (2019). The STEM scale correlated 0.28 with the SRI scale for spatial ability assessment (which only taps into the intrinsic-dynamic quadrant). This means that the nine qualitative statements of the STEM scale correlate with spatial ability at almost comparable levels to more traditional test instruments, whilst simultaneously tapping into a broader spatial ability construct based on the quadrant model. Spatial ability tests typically do not assess three of the quadrants (namely, extrinsic-dynamic, extrinsic-static, and intrinsic-static). However, they are important for real-world applications, such as making activities (Ramey et al., 2020). Therefore, the broad spatial ability construct of the STEM scale could be especially useful for such applications.

Because engagement is associated with achievement and retention in school (Finn and Zimmer, 2012; Moubayed et al., 2020; Piscitello et al., 2022), we also wanted to base the STEM scale on positive engagement as measured by the PSEB scale (Skinner et al., 2009). The correlation was 0.51 when corrected for attenuation and indicates a substantial overlap between the assessed construct by the STEM scale and the PSEB scale. So, while either a very high or very low score on the STEM scale implies both high or low spatial ability and engagement for the student, a more moderate score will mean that either both spatial ability and engagement scores are medium, or that one may be high while the other is low. At first, this may seem problematic, but the differentiation needed for all three combinations may be quite similar due to the interaction between spatial ability and engagement for educational outcomes.

However, if engagement scores are very low and STEM scale scores are quite high concurrently, one could suspect that the student has very high spatial ability. This should be carefully considered as such a lack of engagement is linked to underperforming and risk of school drop-out (Finn and Zimmer, 2012; Piscitello et al., 2022). In this case, although it can be argued that the lower STEM scale score indicates a need for differentiation towards the lower part of the spectrum, somewhat more complex material is needed (Smedsrud et al., 2024). Students could also be given the PSEB subscale to identify their engagement level to disentangle the origin of medium STEM scale scores. Because this is only a five-item scale, it will not be much more time-consuming than giving the nine-item STEM scale to students. Moreover, some countries can have concerns with ability testing, which are omitted by combining the STEM scale and the PSEB subscale instead.

#### 4.2.2 Making activities as practice for differentiation

The developed STEM scale demonstrates good psychometric properties and correlations with spatial ability and engagement as hypothesised. As argued above, it can be used to identify differentiation levels in STEM subjects. Such a differentiation can be done in diverse manners, and we will discuss implications for using making activities due to their strong connection with spatial ability and increased student engagement.

Making activities can be differentiated by varying the openness of the task, i.e., how much structure and scaffolding is provided by the written materials for students (Flø and Zambrana, n.d., In review). These can range from purely open conditions where students explore without boundaries to recipe-like step-by-step walk-throughs of the task. Excluding the extreme points on the scale of the openness of the written materials, the rest of the spectrum can be used as a means of differentiation (Flø and Zambrana, n.d., In review), where those students scoring high on the STEM scale may be provided with somewhat less scaffolded materials. Furthermore, the instruction by teachers can, to a lesser degree, be varied to facilitate differentiation, as it was found that in most cases, even gifted students required teaching of STEM concepts to connect the design and making of the physical artefacts with more theoretical STEM content (Flø and Zambrana, n.d., In review). Proper differentiation may increase feelings of mastery, and as making activities generally increase engagement and enjoyment (Konstantinou et al., 2021), this effect can be reinforced in a making environment. Such an effect can facilitate students’ motivation to engage with more difficult learning material, as ability and motivation interact to predict STEM achievement (Atit et al., 2020). Thus, potentially increasing STEM learning as a result.

Making activities can also act as a way of differentiating for students with higher spatial ability than verbal/mathematical ability, who do not have a particular offer in a more classical school STEM approach (Wai and Uttal, 2018; Wai and Worrell, 2016). These students are more at risk for drop-out, greater academic troubles, and behavioural troubles (Lakin and Wai, 2020), so that the increased valuation of their spatial talents and feelings of mastery induced by proper differentiation may benefit such students. There is also a gender perspective to these students, as one of the most consistent findings regarding gender differences within cognitive science/psychology research is that average male spatial ability test scores are better than the average for females (and vice versa for verbal ability), although this has recently been somewhat contested due to suggested gender bias when constructing mental rotation spatial ability tests (Bartlett and Camba, 2023). However, if these gender differences are not purely bias-caused, they suggest that identifying students with above-average spatial abilities followed up by an intervention with a similar focus could result in a positive impact for males in particular. Because males are overrepresented in several negative life outcome statistics, such as school dropout rates in several countries (Greene and Winters, 2006; Pekkarinen, 2012), increased differentiation in school could benefit engagement, thus reducing school dropout rates. On the other hand, because lower spatial ability can act as a barrier to STEM learning (Dawson, 2019), an intervention targeted towards increasing this ability can positively impact females’ learning and interest in STEM. Differentiated making activities could be an appropriate intervention for both increasing spatial ability and the valuation of such abilities.

Moreover, making activities can increase spatial ability in a curriculum-friendly way so that valuable classroom time is not lost to specific, non-relevant spatial ability interventions. Students also learn STEM from such activities if designed to do so (Falloon et al., 2020; Litts et al., 2017; Peppler and Glosson, 2013). Stieff et al. (2016) argue that interventions targeting representational competence may be advantageous over training generic spatial ability, and another study found that more life-like representations lead to better learning (van der Meij and de Jong, 2006). As making activities consist of working with physical artefacts, i.e., the most life-like representations available, this could be beneficial for STEM learning, as long as representational competencies are addressed as part of the activity. As such, a more naturalistic, contextualised, and educationally relevant spatial ability training would not be time away from the curriculum when spatial ability can be improved through directly STEM-relevant maker activities (Munoz-Rubke et al., 2021), indicating a benefit over a more traditional approach to spatial ability training interventions.

To summarise, the major contribution of the STEM scale to the field is threefold. First, that it correlates with spatial ability at a level almost comparable to spatial ability tests is particularly important for its use in countries where ability testing is uncommon. In such countries, the STEM scale can be used to gain some information on spatial ability and assess differentiation needs, if an engagement scale is used concurrently to control for the engagement part of the STEM scale. The second major contribution of the STEM scale is that it taps into all four quadrants of the quadrant model for spatial ability, which is broader than most other pen-and-paper spatial ability assessments. Because the spatial abilities associated with the less common quadrants are particularly useful in real-life situations, the scale is potentially more useful for such situations than existing instruments. An example of such real-life educational situations is making activities, which seem to be a particularly beneficial type of spatial ability developing intervention in schools. The third major contribution of the STEM scale is its suitability to identify differentiation needs in STEM based on spatial ability, because receiving suitably difficult STEM tasks can facilitate both engagement and achievement for students with higher levels of spatial ability than verbal or mathematical abilities. This is a central contribution because such students are generally more likely to underachieve and drop out of school, and identifying them must be done if they are to receive properly differentiated STEM education.

## 5 Limitations of the study

Regarding the joining of answering categories, this scale is validated based on providing five answering alternatives to students and, in later analyses, joining two of them. Thus, it cannot directly be inferred that these categories can be joined in the scale given to students, and we recommend keeping all five categories for data collection.

Some items may be specific to the Norwegian curriculum for the age group used for validation. Furthermore, the scale is in Norwegian and should thus be validated for other contexts and languages as well. Also, there is a need to broaden the population for which the STEM scale is validated, as the current study validated the scale for 5th to 10th graders.

## 6 Conclusion

The STEM scale demonstrated good psychometric properties, which were aligned with our purpose of tapping into one broad spatial ability construct so that it could be relevant to most STEM subjects and real-life settings. Construct validity was deemed satisfactory based on the scale’s correlations with a spatial ability assessment instrument (the SRI) and one engagement scale (the PSEB). The scale correlated significantly with both, making it a suitable instrument for student differentiation needs, as these needs depend on both the student’s ability level and engagement level. To address the differentiation needs identified by the STEM scale, making activities may be particularly useful due to their general ability to increase engagement and allow students to use their spatial abilities in more ways than what is typical in school, as well as potentially increase spatial ability and STEM learning. Students with lower STEM scale scores could benefit from these activities because they will generally have lower engagement and spatial ability, which can be ameliorated by making activities. Moreover, students with high spatial ability can use their talents by designing and making STEM-related objects, experiencing feelings of mastery, which are central when verbal/mathematical abilities are significantly lower. To further address whether students may increase STEM learning through making activities and whether such learning can be connected to STEM scale scores will be investigated in future work.

The three major contributions of the STEM scale to the field are (1) that it can be used to gain some information on spatial ability levels where such testing is uncommon, (2) that it measures a spatial ability construct more relevant to real-life situations than most other assessments, and (3) that it can be used to identify differentiation needs in STEM to increase retainment and achievement.

## Data Availability

The datasets presented in this study can be found in online repositories. The names of the repository/repositories and accession number(s) can be found at: Mendeley Data Repository (doi: 10.17632/9v6wy5fx3h.1).

## References

[ref1] Amerstorfer C. M. Münster-Kistner C. F. (2021). Student perceptions of academic engagement and student-teacher relationships in problem-based learning. Front. Psychol. 12:713057. doi: 10.3389/fpsyg.2021.713057, PMID: 34777094 PMC8580851

[ref2] Andersen L. (2014). Visual-spatial ability: important in STEM, ignored in gifted education. Roeper Rev. 36, 114–121. doi: 10.1080/02783193.2014.884198

[ref3] Atit K. Power J. R. Pigott T. Lee J. Geer E. A. Uttal D. H.. (2021). Examining the relations between spatial skills and mathematical performance: a meta-analysis. Psychon. Bull. Rev. 29, 699–720. doi: 10.3758/s13423-021-02012-w, PMID: 34799844

[ref4] Atit K. Power J. R. Veurink N. Uttal D. H. Sorby S. Panther G.. (2020). Examining the role of spatial skills and mathematics motivation on middle school mathematics achievement. Int. J. STEM Educ. 7, 1–13.

[ref5] Bartlett K. A. Camba J. D. (2023). Gender differences in spatial ability: a critical review. Educ. Psychol. Rev. 35:8. doi: 10.1007/s10648-023-09728-2

[ref7] Bhaduri S. Van Horne K. Sumner T. (2019). *Designing an informal learning curriculum to develop 3D modeling knowledge and improve spatial thinking skills*. In Extended abstracts of the 2019 CHI conference on human factors in computing systems. 1–8.

[ref8] Boateng G. O. Neilands T. B. Frongillo E. A. Melgar-Quiñonez H. R. Young S. L. (2018). Best practices for developing and validating scales for health, social, and behavioral research: a primer. Front. Public Health 6:149. doi: 10.3389/fpubh.2018.00149, PMID: 29942800 PMC6004510

[ref9] Bock R. D. Mislevy R. J. (1982). Adaptive EAP estimation of ability in a microcomputer environment. Appl. Psychol. Meas. 6, 431–444. doi: 10.1177/014662168200600405

[ref10] Bongers A. Beauvoir B. Streja N. Northoff G. Flynn A. B. (2020). Building mental models of a reaction mechanism: the influence of static and animated representations, prior knowledge, and spatial ability. Chem. Educ. Res. Pract. 21, 496–512. doi: 10.1039/C9RP00198K

[ref9003] Brevik L. M. Gunnulfsen A. E. and Renzulli J. S. (2018). Student teachers’ practice and experience with differentiated instruction for students with higher learning potential. Teach. Teach. Educ, 71, 34–45.

[ref11] Brown A. (2018). “Item response theory approaches to test scoring and evaluating the score accuracy” in The Wiley handbook of psychometric testing: A multidisciplinary reference on survey, scale and test development. eds. Irwing P. Booth T. Hughes D. J. (New York: John Wiley & Sons), 607–638.

[ref12] Buckley J. Seery N. Canty D. (2018). A heuristic framework of spatial ability: a review and synthesis of spatial factor literature to support its translation into STEM education. Educ. Psychol. Rev. 30, 947–972. doi: 10.1007/s10648-018-9432-z

[ref13] Buckley J. Seery N. Canty D. Gumaelius L. (2022). “The importance of spatial ability within technology education” in Applications of research in technology education: Helping teachers develop research-informed practice. ed. Buckley J. (Singapore: Springer Nature Singapore), 165–182.

[ref14] Cai L. Monroe S. (2014). *A new statistic for evaluating item response theory models for ordinal data (CRESST Report 839)*. Available online at: https://files.eric.ed.gov/fulltext/ED555726.pdf.

[ref15] Carpenter S. (2018). Ten steps in scale development and reporting: a guide for researchers. Commun. Methods Meas. 12, 25–44. doi: 10.1080/19312458.2017.1396583

[ref16] Castro-Alonso J. C. (2019). “Overview of visuospatial processing for education in health and natural sciences” in Visuospatial processing for education in health and natural sciences. eds. Castro-Alonso J. C. Castro-Alonso J. C. (Cham: Springer), 1–21.

[ref17] Castro-Alonso J. C. Atit K. (2019). “Different abilities controlled by visuospatial processing” in Visuospatial processing for education in health and natural sciences. ed. Castro-Alonso J. C. (Cham: Springer), 23–51.

[ref18] Chalmers R. P. (2012). Mirt: a multidimensional item response theory package for the R environment. J. Stat. Softw. 48, 1–29. doi: 10.18637/jss.v048.i06

[ref19] Chen Y. C. Yang F. Y. Chang C. C. (2020). Conceptualizing spatial abilities and their relation to science learning from a cognitive perspective. J. Balt. Sci. Educ. 19, 50–63. doi: 10.33225/jbse/20.19.50

[ref20] Cheng Y. Yuan K.-H. Liu C. (2012). Comparison of reliability measures under factor analysis and item response theory. Educ. Psychol. Meas. 72, 52–67. doi: 10.1177/0013164411407315

[ref21] Cho S. J. Suh Y. Lee W. Y. (2016). An NCME instructional module on latent DIF analysis using mixture item response models. Educ. Meas. Issues Pract. 35, 48–61. doi: 10.1111/emip.12093

[ref22] Cortes R. A. Peterson E. G. Kraemer D. J. Kolvoord R. A. Uttal D. H. Dinh N.. (2022). Transfer from spatial education to verbal reasoning and prediction of transfer from learning-related neural change. Sci. Adv. 8:eabo3555. doi: 10.1126/sciadv.abo3555, PMID: 35947663 PMC9365289

[ref23] Crocker L. Algina J. (1986). Introduction to classical and modern test theory. Orlando, FL: Holt, Rinehart and Winston.

[ref24] Dawson C. (2019). Tackling limited spatial ability: lowering one barrier into STEM? Eur. J. Sci. Math. Educ. 7, 14–31. doi: 10.30935/scimath/9531

[ref9002] Diezmann C. (2005). Challenging mathematically gifted primary students. Australas. J. Gift. Educ, 14, 50–57.

[ref25] Edelen M. O. Reeve B. B. (2007). Applying item response theory (IRT) modeling to questionnaire development, evaluation, and refinement. Qual. Life Res. 16 Suppl 1, 5–18. doi: 10.1007/s11136-007-9198-0, PMID: 17375372

[ref26] Ertl B. Luttenberger S. Paechter M. (2017). The impact of gender stereotypes on the self-concept of female students in STEM subjects with an under-representation of females. Front. Psychol. 8:703. doi: 10.3389/fpsyg.2017.00703, PMID: 28567022 PMC5434750

[ref27] Falloon G. Forbes A. Stevenson M. Bower M. Hatzigianni M. (2020). STEM in the making? Investigating STEM learning in junior school makerspaces. Res. Sci. Educ. 52, 511–537. doi: 10.1007/s11165-020-09949-3

[ref28] Finn J. D. Zimmer K. S. (2012). “Student engagement: what is it? Why does it matter?” in Handbook of research on student engagement. eds. Christenson S. L. Reschly A. L. Wylie C. (Berlin: Springer), 97–131.

[ref31] Fredricks J. A. Filsecker M. Lawson M. A. (2016). Student engagement, context, and adjustment: addressing definitional, measurement, and methodological issues. Learn. Instr. 43, 1–4. doi: 10.1016/j.learninstruc.2016.02.002

[ref32] Gander F. Proyer R. T. Ruch W. (2016). Positive psychology interventions addressing pleasure, engagement, meaning, positive relationships, and accomplishment increase well-being and ameliorate depressive symptoms: a randomized, placebo-controlled online study. Front. Psychol. 7:686. doi: 10.3389/fpsyg.2016.00686, PMID: 27242600 PMC4873493

[ref33] González-Pérez S. Mateos de Cabo R. Sáinz M. (2020). Girls in STEM: is it a female role-model thing? Front. Psychol. 11:564148. doi: 10.3389/fpsyg.2020.02204 PMC7511552 33013573

[ref34] Gorsuch R. L. Venable G. D. (1983). Development of an" age universal" IE scale. J. Sci. Study Relig 22, 181–187. doi: 10.2307/1385677

[ref35] Greene J. P. Winters M. A. (2006). *Leaving boys behind: Public high school graduation rates*. Education Working Paper Archive.

[ref36] Groccia J. E. (2018). What is student engagement? New Dir. Teach. Learn. 2018, 11–20. doi: 10.1002/tl.20287

[ref37] Guo S. Wang X. Deng W. Hong J. Wang J. Wu Y. (2022). Whose spatial ability benefits from learning with 3D design? From the perspective of learning analysis. Educ. Technol. Soc. 25, 179–192.

[ref38] Hambleton R. K. Cook L. L. (1977). Latent trait models and their use in the analysis of educational test data. J. Educ. Meas. 1, 75–96.

[ref39] Harris D. Logan T. Lowrie T. (2021). Unpacking mathematical-spatial relations: problem-solving in static and interactive tasks. Math. Educ. Res. J. 33, 495–511. doi: 10.1007/s13394-020-00316-z

[ref40] Hawes Z. Tepylo D. Moss J. (2015). *Developing spatial reasoning*. Spatial reasoning in the early years, pp. 29–44.

[ref41] Hodgkiss A. Gilligan K. A. Tolmie A. K. Thomas M. S. C. Farran E. K. (2018). Spatial cognition and science achievement: the contribution of intrinsic and extrinsic spatial skills from 7 to 11 years. Br. J. Educ. Psychol. 88, 675–697. doi: 10.1111/bjep.12211, PMID: 29359476 PMC6283002

[ref42] Kalinowski S. T. (2019). A graphical method for displaying the model fit of item response theory trace lines. Educ. Psychol. Meas. 79, 1064–1074. doi: 10.1177/0013164419846234, PMID: 31619840 PMC6777066

[ref43] Kang T. Chen T. T. (2008). Performance of the generalized S-X2 item fit index for polytomous IRT models. J. Educ. Meas. 45, 391–406. doi: 10.1111/j.1745-3984.2008.00071.x

[ref44] Kang T. Chen T. T. (2011). Performance of the generalized SX 2 item fit index for the graded response model. Asia Pac. Educ. Rev. 12, 89–96. doi: 10.1007/s12564-010-9082-4

[ref45] Kell H. J. Lubinski D. Benbow C. P. Steiger J. H. (2013). Creativity and technical innovation: spatial ability’s unique role. Psychol. Sci. 24, 1831–1836. doi: 10.1177/0956797613478615, PMID: 23846718

[ref46] Kim S.-H. Cohen A. S. Cho S.-J. Eom H. J. (2019). Use of information criteria in the study of group differences in trace lines. Appl. Psychol. Meas. 43, 95–112. doi: 10.1177/0146621618772292, PMID: 30792558 PMC6376536

[ref47] Kong Y. (2021). The role of experiential learning on students’ motivation and classroom engagement. Front. Psychol. 12:771272. doi: 10.3389/fpsyg.2021.771272, PMID: 34744950 PMC8569223

[ref48] Konstantinou D. Parmaxi A. Zaphiris P. (2021). Mapping research directions on makerspaces in education. Educ. Media Int. 58, 223–247. doi: 10.1080/09523987.2021.1976826

[ref49] Kozhevnikov M. Hegarty M. (2001). A dissociation between object manipulation spatial ability and spatial orientation ability. Mem. Cogn. 29, 745–756. doi: 10.3758/BF03200477, PMID: 11531229

[ref50] Lakin J. M. Wai J. (2020). Spatially gifted, academically inconvenienced: spatially talented students experience less academic engagement and more behavioural issues than other talented students. Br. J. Educ. Psychol. 90, 1015–1038. doi: 10.1111/bjep.12343, PMID: 32065397

[ref51] Lavonen J. Laaksonen S. (2009). Context of teaching and learning school science in Finland: reflections on PISA 2006 results. J. Res. Sci. Teach. 46, 922–944. doi: 10.1002/tea.20339

[ref52] Levine S. C. Foley A. Lourenco S. Ehrlich S. Ratliff K. (2016). Sex differences in spatial cognition: advancing the conversation. Wiley Interdiscip. Rev. Cogn. Sci. 7, 127–155. doi: 10.1002/wcs.1380, PMID: 26825049

[ref53] Li X. Wang W. (2021). Exploring spatial cognitive process among STEM students and its role in STEM education. Sci. & Educ. 30, 1–25. doi: 10.1007/s11191-020-00167-x

[ref54] Linn M. C. Petersen A. C. (1985). Emergence and characterization of sex differences in spatial ability: a meta-analysis. Child Dev. 56, 1479–1498. doi: 10.2307/1130467, PMID: 4075870

[ref55] Litts B. K. Kafai Y. B. Lui D. A. Walker J. T. Widman S. A. (2017). Stitching codeable circuits: high school students’ learning about circuitry and coding with electronic textiles. J. Sci. Educ. Technol. 26, 494–507. doi: 10.1007/s10956-017-9694-0

[ref56] Liu Y. Tian W. Xin T. (2016). An application of M 2 statistic to evaluate the fit of cognitive diagnostic models. J. Educ. Behav. Stat. 41, 3–26. doi: 10.3102/1076998615621293

[ref57] Lohman D. F. (2013). “Spatial ability and g” in Human abilities. ed. Lohman D. F. (Hove, East Sussex: Psychology Press), 97–116.

[ref58] Lowrie T. Logan T. Ramful A. (2017). Visuospatial training improves elementary students’ mathematics performance. Br. J. Educ. Psychol. 87, 170–186. doi: 10.1111/bjep.12142, PMID: 28097646

[ref59] Malanchini M. Rimfeld K. Shakeshaft N. G. McMillan A. Schofield K. L. Rodic M.. (2020). Evidence for a unitary structure of spatial cognition beyond general intelligence. NPJ Sci. Learn. 5:9. doi: 10.1038/s41539-020-0067-8, PMID: 32655883 PMC7331750

[ref60] Maxwell S. Reynolds K. J. Lee E. Subasic E. Bromhead D. (2017). The impact of school climate and school identification on academic achievement: multilevel modeling with student and teacher data. Front. Psychol. 8:2069. doi: 10.3389/fpsyg.2017.02069, PMID: 29259564 PMC5723344

[ref61] Maydeu-Olivares A. (2013). Goodness-of-fit assessment of item response theory models. Meas. Interdiscip. Res. Perspect. 11, 71–101. doi: 10.1080/15366367.2013.831680

[ref62] Maydeu-Olivares A. Joe H. (2006). Limited information goodness-of-fit testing in multidimensional contingency tables. Psychometrika 71, 713–732. doi: 10.1007/s11336-005-1295-9

[ref63] Mercan Z. Kandır A. (2022). The effect of the early STEAM education program on the visual-spatial reasoning skills of children: research from Turkey. Education 3–13 52, 123–153. doi: 10.1080/03004279.2022.2075906

[ref64] Mersand S. (2021). The state of makerspace research: a review of the literature. TechTrends 65, 174–186. doi: 10.1007/s11528-020-00566-5

[ref65] Mohler J. L. (2008). A review of spatial ability research. Eng. Des. Graph. J. 1, 19–30.

[ref66] Moubayed A. Injadat M. Shami A. Lutfiyya H. (2020). Student engagement level in an e-learning environment: clustering using K-means. Am. J. Distance Educ. 34, 137–156. doi: 10.1080/08923647.2020.1696140

[ref67] Munoz-Rubke F. Will R. Hawes Z. James K. H. (2021). Enhancing spatial skills through mechanical problem solving. Learn. Instr. 75:101496. doi: 10.1016/j.learninstruc.2021.101496

[ref68] National Research Council and Geographical Sciences Committee (2005). Learning to think spatially. Washington, DC: National Academies Press.

[ref69] Newcombe N. (2017). Harnessing spatial thinking to support stem learning. Paris, France: OCED, 51.

[ref70] Newcombe N. S. Shipley T. F. (2015). “Thinking about spatial thinking: new typology, new assessments” in Studying visual and spatial reasoning for design creativity. ed. Gero J. S. (New York, NY: Springer), 179–192.

[ref71] Norwegian Directorate for Education and Vocational Training. (2018). *Notat om programmering i skolen*. Available online at: https://www.udir.no/globalassets/filer/programmering_i_skolen.pdf (Accessed September 11, 2022).

[ref72] Pekkarinen T. (2012). Gender differences in education. Nord. Econ. Policy Rev. 1, 165–194.

[ref73] Peppler K. Glosson D. (2013). Stitching circuits: learning about circuitry through e-textile materials. J. Sci. Educ. Technol. 22, 751–763. doi: 10.1007/s10956-012-9428-2

[ref74] Piscitello J. Kim Y. K. Orooji M. Robison S. (2022). Sociodemographic risk, school engagement, and community characteristics: a mediated approach to understanding high school dropout. Child Youth Serv. Rev. 133:106347. doi: 10.1016/j.childyouth.2021.106347

[ref75] Plummer J. D. Udomprasert P. Vaishampayan A. Sunbury S. Cho K. Houghton H.. (2022). Learning to think spatially through curricula that embed spatial training. J. Res. Sci. Teach. 59, 1134–1168. doi: 10.1002/tea.21754

[ref76] R Core Team. (2023). *R: A language and environment for statistical computing [Computer software]*. R Foundation for Statistical Computing Available online at: https://www.R-project.org/.

[ref77] Ramey K. E. Stevens R. Uttal D. H. (2020). In-FUSE-ing STEAM learning with spatial reasoning: distributed spatial sensemaking in school-based making activities. J. Educ. Psychol. 112, 466–493. doi: 10.1037/edu0000422

[ref78] Ramful A. Lowrie T. Logan T. (2017). Measurement of spatial ability: construction and validation of the spatial reasoning instrument for middle school students. J. Psychoeduc. Assess. 35:59207. doi: 10.1177/0734282916659207

[ref79] Rios J. Wells C. (2014). Validity evidence based on internal structure. Psicothema 26, 108–116. doi: 10.7334/psicothema2013.260, PMID: 24444738

[ref9005] Ritoša A. Danielsson H. Sjöman M. Almqvist L. Granlund M. (2020). Assessing school engagement–adaptation and validation of “engagement versus disaffection with learning: Teacher report” in the Swedish educational context. In Front. Educ. Frontiers Media SA. 5: 521972.

[ref9004] Rotigel J. V. Fello S. (2004). Mathematically gifted students: How can we meet their needs?. Gift. Child Today, 27, 46–51.

[ref80] Salas-Pilco S. Z. Yang Y. Zhang Z. (2022). Student engagement in online learning in Latin American higher education during the COVID-19 pandemic: a systematic review. Br. J. Educ. Technol. 53, 593–619. doi: 10.1111/bjet.13190, PMID: 35600418 PMC9111674

[ref81] Schmidt M. Benzing V. Kamer M. (2016). Classroom-based physical activity breaks and children's attention: cognitive engagement works! Front. Psychol. 7:1474. doi: 10.3389/fpsyg.2016.01474, PMID: 27757088 PMC5047899

[ref82] Simpson A. Kastberg S. (2022). Makers do math! Legitimizing informal mathematical practices within making contexts. J. Humanist. Math. 12, 40–75. doi: 10.5642/jhummath.202201.05

[ref83] Skinner E. A. Kindermann T. A. Furrer C. J. (2009). A motivational perspective on engagement and disaffection: conceptualization and assessment of children's behavioral and emotional participation in academic activities in the classroom. Educ. Psychol. Meas. 69, 493–525. doi: 10.1177/0013164408323233

[ref84] Smedsrud J. (2018). Mathematically gifted accelerated students participating in an ability group: a qualitative interview study. Front. Psychol. 9:1359. doi: 10.3389/fpsyg.2018.01359, PMID: 30108542 PMC6080139

[ref85] Smedsrud J. H. Bungum B. Flø E. E. (2024). Gifted students’ experiences with participation in enrichment programs at talent centers in Norway. Scand. J. Educ. Res. 69, 1080–1096. doi: 10.1080/00313831.2024.2394388

[ref86] Smith S. (2018). Children’s negotiations of visualization skills during a design-based learning experience using nondigital and digital techniques. Interdiscip. J. Probl. Based Learn. 12:4. doi: 10.7771/1541-5015.1747, PMID: 29356800

[ref87] Sorby S. Veurink N. Streiner S. (2018). Does spatial skills instruction improve STEM outcomes? The answer is ‘yes’. Learn. Individ. Differ. 67, 209–222. doi: 10.1016/j.lindif.2018.09.001

[ref9001] Steenbergen-Hu S. Olszewski-Kubilius P. Calvert E. (2020). The effectiveness of current interventions to reverse the underachievement of gifted students: Findings of a meta-analysis and systematic review. Gift. Child Q. 64, 132–165.

[ref88] Stieff M. Origenes A. DeSutter D. Lira M. Banevicius L. Tabang D.. (2018). Operational constraints on the mental rotation of STEM representations. J. Educ. Psychol. 110, 1160–1174. doi: 10.1037/edu0000258

[ref89] Stieff M. Scopelitis S. Lira M. E. Desutter D. (2016). Improving representational competence with concrete models. Sci. Educ. 100, 344–363. doi: 10.1002/sce.21203

[ref90] Tanenbaum C. (2016). STEM 2026: A vision for innovation in STEM education. Washington, DC: US Department of Education.

[ref91] Trumble J. Dailey D. (2019). Change in spatial visualization mental rotation abilities of intermediate elementary students. J. Comput. Math. Sci. Teach. 38, 77–90. doi: 10.70725/897929paonwo

[ref92] Uttal D. H. Meadow N. G. Tipton E. Hand L. L. Alden A. R. Warren C.. (2013). The malleability of spatial skills: a meta-analysis of training studies. Psychol. Bull. 139, 352–402. doi: 10.1037/a0028446, PMID: 22663761

[ref93] van der Meij J. de Jong T. (2006). Supporting students' learning with multiple representations in a dynamic simulation-based learning environment. Learn. Instr. 16, 199–212. doi: 10.1016/j.learninstruc.2006.03.007

[ref94] Wai J. Lubinski D. Benbow C. P. (2009). Spatial ability for STEM domains: aligning over 50 years of cumulative psychological knowledge solidifies its importance. J. Educ. Psychol. 101, 817–835. doi: 10.1037/a0016127

[ref95] Wai J. Uttal D. H. (2018). Why spatial reasoning matters for education policy. Washington, DC: American Enterprise Institute.

[ref96] Wai J. Worrell F. C. (2016). Helping disadvantaged and spatially talented students fulfill their potential: related and neglected national resources. Policy Insights Behav. Brain Sci. 3, 122–128. doi: 10.1177/2372732215621310

[ref97] Young M. Muller J. (2016). Curriculum and the specialization of knowledge. Abingdon: Routledge.

[ref98] Zeng G. Hou H. Peng K. (2016). Effect of growth mindset on school engagement and psychological well-being of Chinese primary and middle school students: the mediating role of resilience. Front. Psychol. 7:1873. doi: 10.3389/fpsyg.2016.01873, PMID: 28018251 PMC5147462

