# Supplementary material for: Assessing STEM differentiation needs based on spatial ability and engagement: implications for making activities
Source: Front Psychol. 2025 Aug 26;16:1545603. doi: 10.3389/fpsyg.2025.1545603 (PMC12419082; doi:10.3389/fpsyg.2025.1545603)
Supplement: Supplementary file 1 [file Data_Sheet_1.pdf]

Appendix A

1. I am good at understanding coordinate systems.
2. I am good at finding what is important in tables or graphs.
3. It is easy for me to find what is important in a text.
4. I understand what different molecules look like.
5. I can easily understand the connection between a fraction and the number line.
6. I am good at understanding the connection between a graph and its corresponding formula.
7. I am good at making the right connections in electrical circuits.
8. I understand why the backside of the moon is always facing away from the earth.
9. I am good at understanding what we are supposed to do in science experiments.

I remember best what is in a textbook based on the pictures and figures in the text.

It is easy to remember the figures and images in science.

It is easy to understand the figures and images in science.

I am good at jigsaw puzzles or assembling things.

I am good at assembling advanced building sets or IKEA furniture.

I am good at taking things apart and then putting them back together again.

I am good at telling the difference between right and left.

I am good at following directions.

I am good at understanding maps.

I am good at finding my way through a crowd.

I am better than others at finding things when I'm looking for them.

I am good at finding answers in schoolbooks or on the internet.

I understand the connection between the seasons and the fact that the Earth tilts as it orbits the Sun.
